# {4-Hy­droxy-*N*′-[(2-oxido-1-naphthyl-κ*O*)methyl­idene]benzohydrazidato-κ^2^ *N*′,*O*}dimethyl­tin(IV)

**DOI:** 10.1107/S1600536810027078

**Published:** 2010-07-14

**Authors:** Md. Abu Affan, Norrihan B. Sam, Fasihuddin B. Ahmad, Edward R. T. Tiekink

**Affiliations:** a Faculty of Resource Science and Technology, Universiti Malaysia Sarawak, 94300 Kota Samarahan, Sarawak, Malaysia; b Department of Chemistry, University of Malaya, 50603 Kuala Lumpur, Malaysia

## Abstract

Two independent but very similar mol­ecules comprise the asymmetric unit of the title compound, [Sn(CH_3_)_2_(C_18_H_12_N_2_O_3_)]. Each Sn atom is coordinated by two methyl groups and two O atoms and an N atom from the dinegative tridentate ligand. The resultant C_2_NO_2_ donor set defines a coordination geometry inter­mediate between square-pyramidal and trigonal-pyramidal, with a small tendency towards the former. Zigzag chains running along the *a* axis mediated by O—H⋯N hydrogen bonding characterize the crystal packing. These are connected into layers in the *ab* plane by a combination of C—H⋯N and π–π [centroid–centroid distances = 3.658 (2) and 3.6740 (18) Å] inter­actions. The layers are connected along the *c* axis *via* C—H⋯O inter­actions.

## Related literature

For related studies on organotin compounds, see: Affan *et al.* (2009 ▶); Zukerman-Schpector *et al.* (2009 ▶). For the structure of the dichloro­methane solvate of the title compound, see: Cui *et al.* (2007 ▶). For coordination geometry, see: Addison *et al.* (1984 ▶).
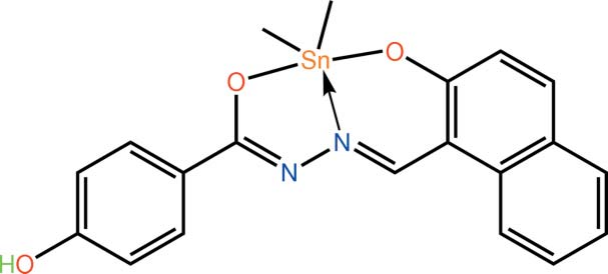


## Experimental

### 

#### Crystal data

[Sn(CH_3_)_2_(C_18_H_12_N_2_O_3_)]
*M* *_r_* = 453.05
Monoclinic, 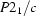

*a* = 12.9422 (4) Å
*b* = 16.5264 (5) Å
*c* = 16.9949 (5) Å
β = 94.923 (3)°
*V* = 3621.59 (19) Å^3^
*Z* = 8
Mo *K*α radiation
μ = 1.43 mm^−1^
*T* = 150 K
0.20 × 0.15 × 0.06 mm

#### Data collection

Oxford Diffraction Gemini E diffractometer
Absorption correction: multi-scan (*CrysAlis PRO*; Oxford Diffraction, 2010 ▶) *T*_min_ = 0.863, *T*_max_ = 1.000
12413 measured reflections
6668 independent reflections
5647 reflections with *I* > 2σ(*I*)
*R*_int_ = 0.027

#### Refinement

*R*[*F* ^2^ > 2σ(*F* ^2^)] = 0.031
*wR*(*F* ^2^) = 0.069
*S* = 1.05
6668 reflections
475 parameters
H-atom parameters constrained
Δρ_max_ = 0.73 e Å^−3^
Δρ_min_ = −0.51 e Å^−3^

### 

Data collection: *CrysAlis PRO* (Oxford Diffraction, 2010 ▶); cell refinement: *CrysAlis PRO*; data reduction: *CrysAlis PRO*; program(s) used to solve structure: *SHELXS97* (Sheldrick, 2008 ▶); program(s) used to refine structure: *SHELXL97* (Sheldrick, 2008 ▶); molecular graphics: *ORTEP-3* (Farrugia, 1997 ▶) and *DIAMOND* (Brandenburg, 2006 ▶); software used to prepare material for publication: *publCIF* (Westrip, 2010 ▶).

## Supplementary Material

Crystal structure: contains datablocks global, I. DOI: 10.1107/S1600536810027078/bt5292sup1.cif

Structure factors: contains datablocks I. DOI: 10.1107/S1600536810027078/bt5292Isup2.hkl

Additional supplementary materials:  crystallographic information; 3D view; checkCIF report

## Figures and Tables

**Table 1:** Hydrogen-bond geometry (Å, °)

*D*—H⋯*A*	*D*—H	H⋯*A*	*D*⋯*A*	*D*—H⋯*A*
O3—H3⋯N3^i^	0.84	1.91	2.749 (3)	178
O6—H6a⋯N1^ii^	0.84	1.91	2.738 (3)	167
C8—H8⋯O5^iii^	0.95	2.59	3.477 (4)	155
C21—H21a⋯N1^iv^	0.98	2.62	3.494 (4)	149

Symmetry codes: (i) 
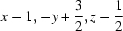
; (ii) 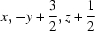; (iii) 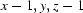; (iv) 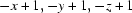.

**Fig. 1.**
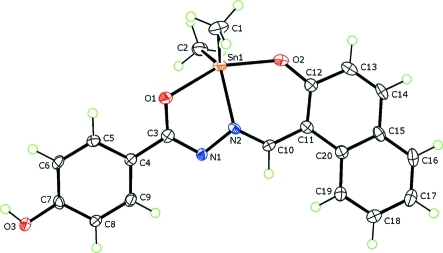
The molecular structure of the first independent molecule of (I) showing the atom-labelling scheme and displacement ellipsoids at the 50% probability level.

**Fig. 2.**
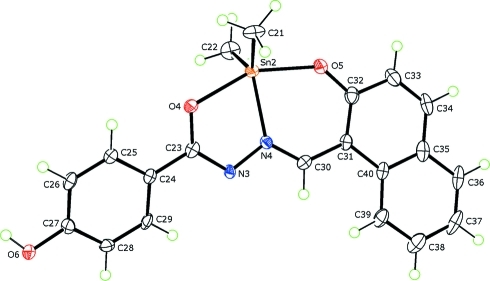
The molecular structure of the second independent molecule of (I) showing the atom-labelling scheme and displacement ellipsoids at the 50% probability level.

**Fig. 3.**
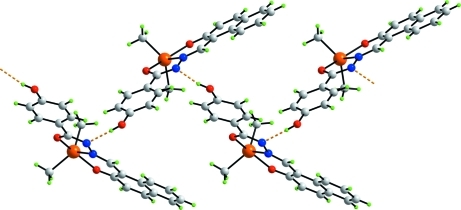
A view of the supramolecular chain along the a axis in (I) mediated by O–H···N hydrogen bonding shown as orange dashed lines.

**Fig. 4.**
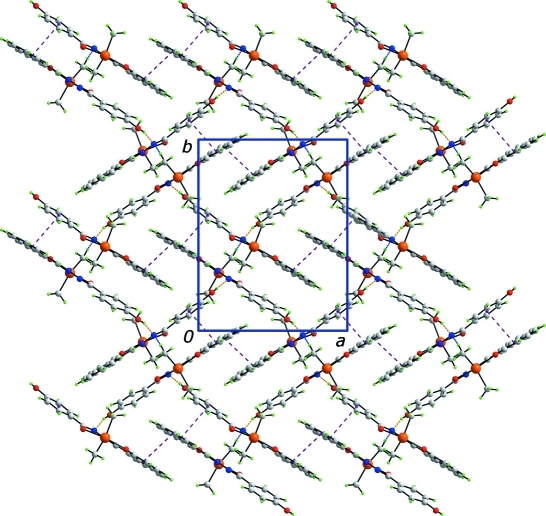
A view of the 2-D supramolecular array in the ab plane in (I) with the O–H···N hydrogen bonding, C–H···N and π–π contacts shown as orange, blue and purple dashed lines, respectively.

**Fig. 5.**
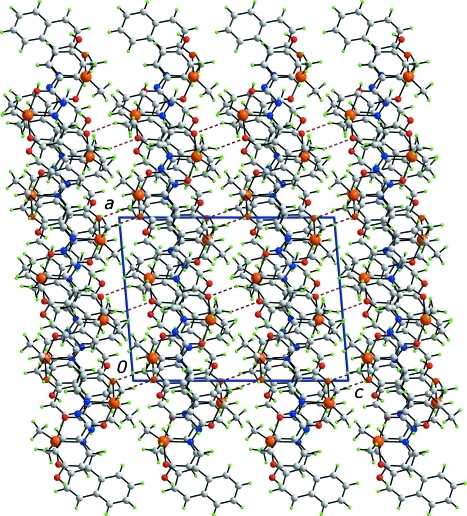
A view in projection down the a axis of (I) showing the stacking of 2-D arrays along the c axis. The C–H···O contacts shown as brown dashed lines.

## supplementary crystallographic information

### Comment

Interest in the title compound (I) stems from on-going studies into biological and structural aspects of organotin compounds (Affan *et al.*, 2009; Zukerman-Schpector *et al.*, 2009). Two independent molecules comprise the crystallographic asymmetric unit of (I) with the first molecule, Fig. 1, being virtually superimposable upon the second, Fig. 2, there being only small differences in the relative orientations of aromatic rings in the molecules. The similarity between the molecules is reflected in r.m.s. values for bond distances and angles of 0.0063 Å and 1.128 °, respectively. The Sn atom environment in each case is based on a C_2_NO_2_ donor set provided by two methyl groups, and the NO_2_ atoms of the dinegative, tridentate ligand. The coordination geometry is intermediate between square pyramidal and trigonal bipyramidal with a leaning towards the former. These assignments are based on the values calculated for τ of 0.48 and 0.43 for the Sn1 and Sn2 atoms, respectively, which compare to the τ values of 0.0 and 1.0 for ideal square pyramidal and trigonal bipyramidal geometries, respectively (Addison *et al.*, 1984).

The crystal packing is dominated by O–H···N hydrogen bonding between the hydroxyl group and the non-coordinating imine-N atoms, Table 1. These lead to the formation of zigzag supramolecular chains along the *a* axis, Fig. 3. Chains are consolidated into layers *via* a combination of C–H···N interactions, Table 1, and π–π contacts, Fig. 4. The latter occur between centrosymmetrically related rings involving both the benzene ring and the fused ring systems, *i.e. Cg*(C11–C15,C20)···*Cg*(C35–C40)^i^ = 3.658 (2) Å and *Cg*(C4–C9)···*Cg*(C31–C35,C40)^ii^ = 3.6740 (18) Å for *i*: 2 - *x*, 1 - *y*, 1 - *z*, and *ii*: 1 - *x*, 1 - *y*, 1 - *z*. Layers thus formed  stack along the *c* axis, being connected by C–H···O contacts, Table 1 and Fig. 5.

The molecular structures in (I) resemble closely that found in the dichloromethane solvate (Cui *et al.*, 2007), which also has an intermediate coordination geometry but slightly distorted to trigonal bipyramidal (τ = 0.51). Interestingly, despite the presence of lattice solvent, the supramolecular zigzag chains mediated by O–H···N hydrogen bonding persist in the literature structure.

### Experimental

2-Hydroxy-1-naphthaldehyde-4-hydroxybenzhydrazone (0.612 g, 2 mmol) was dissolved in hot absolute methanol (20 ml) in a Schlenk round bottom flask under purified nitrogen atmosphere. Then, a potassium hydroxide solution (0.224 g, 4 mmol) in absolute methanol (5 ml) was added dropwise and the colour of the resulting solution faded from yellow to light-yellow. The resulting solution was refluxed under a nitrogen atmosphere for 1 h. A solution of dimethyltin(IV) dichloride (0.439 g, 2 mmol) in absolute methanol (5 ml) was added dropwise. The resulting solution was refluxed for 4 h and allowed to cool to room temperature for 30 min. The white precipitate of potassium chloride was removed by filtration. The filtrate was evaporated to dryness using a rotary apparatus under reduced pressure. The yellow crystals obtained were washed with hexane and dried *in vacuo* over silica gel. Single crystals suitable for X-ray analyses were obtained from the slow evaporation of an ethanol solution at room temperature. Yield: 0.67 g, 53%, and m.pt.540–542 K.

### Refinement

H atoms were geometrically placed (O–H = 0.84 Å and C–H = 0.95–0.98 Å) and refined as riding with *U*_iso_(H) = 1.5*U*_eq_(O) and *U*_iso_(H) = 1.2–1.5*U*_eq_(C).

### Crystal data

**Table:**

[Sn(CH_3_)_2_(C_18_H_12_N_2_O_3_)]	*F*(000) = 1808
*M**_r_* = 453.05	*D*_x_ = 1.662 Mg m^−^^3^
Monoclinic, *P*2_1_/*c*	Mo *K*α radiation, λ = 0.71073 Å
Hall symbol: -P 2ybc	Cell parameters from 7047 reflections
*a* = 12.9422 (4) Å	θ = 2.3–27.4°
*b* = 16.5264 (5) Å	µ = 1.43 mm^−^^1^
*c* = 16.9949 (5) Å	*T* = 150 K
β = 94.923 (3)°	Prism, pink
*V* = 3621.59 (19) Å^3^	0.20 × 0.15 × 0.06 mm
*Z* = 8	

### Data collection

**Table:**

Oxford Diffraction Gemini E diffractometer	6668 independent reflections
Radiation source: fine-focus sealed tube	5647 reflections with *I* > 2σ(*I*)
graphite	*R*_int_ = 0.027
Detector resolution: 10.5081 pixels mm^-1^	θ_max_ = 25.5°, θ_min_ = 2.3°
ω scans	*h* = −15→8
Absorption correction: multi-scan (*CrysAlis PRO*; Oxford Diffraction, 2010)	*k* = −19→19
*T*_min_ = 0.863, *T*_max_ = 1.000	*l* = −20→19
12413 measured reflections	

### Refinement

**Table:**

Refinement on *F*^2^	Primary atom site location: structure-invariant direct methods
Least-squares matrix: full	Secondary atom site location: difference Fourier map
*R*[*F*^2^ > 2σ(*F*^2^)] = 0.031	Hydrogen site location: inferred from neighbouring sites
*wR*(*F*^2^) = 0.069	H-atom parameters constrained
*S* = 1.05	*w* = 1/[σ^2^(*F*_o_^2^) + (0.0238*P*)^2^ + 0.8497*P*] where *P* = (*F*_o_^2^ + 2*F*_c_^2^)/3
6668 reflections	(Δ/σ)_max_ = 0.001
475 parameters	Δρ_max_ = 0.73 e Å^−^^3^
0 restraints	Δρ_min_ = −0.51 e Å^−^^3^

### Special details

**Table:**

Geometry. All s.u.'s (except the s.u. in the dihedral angle between two l.s. planes) are estimated using the full covariance matrix. The cell s.u.'s are taken into account individually in the estimation of s.u.'s in distances, angles and torsion angles; correlations between s.u.'s in cell parameters are only used when they are defined by crystal symmetry. An approximate (isotropic) treatment of cell s.u.'s is used for estimating s.u.'s involving l.s. planes.
Refinement. Refinement of *F*^2^ against ALL reflections. The weighted *R*-factor *wR* and goodness of fit *S* are based on *F*^2^, conventional *R*-factors *R* are based on *F*, with *F* set to zero for negative *F*^2^. The threshold expression of *F*^2^ > 2σ(*F*^2^) is used only for calculating *R*-factors(gt) *etc*. and is not relevant to the choice of reflections for refinement. *R*-factors based on *F*^2^ are statistically about twice as large as those based on *F*, and *R*- factors based on ALL data will be even larger.

### Fractional atomic coordinates and isotropic or equivalent isotropic displacement parameters (Å^2^)

**Table:**

	*x*	*y*	*z*	*U*_iso_*/*U*_eq_	
Sn1	0.372807 (18)	0.439312 (13)	0.384468 (13)	0.01894 (7)	
O1	0.22567 (17)	0.48447 (13)	0.33302 (12)	0.0229 (5)	
O2	0.51368 (18)	0.37879 (14)	0.38188 (13)	0.0280 (6)	
O3	−0.09090 (17)	0.69902 (13)	0.11649 (13)	0.0226 (5)	
H3	−0.1239	0.7217	0.1508	0.034*	
N1	0.2992 (2)	0.47633 (15)	0.21448 (15)	0.0159 (6)	
N2	0.3766 (2)	0.43266 (15)	0.25829 (15)	0.0158 (6)	
C1	0.3054 (3)	0.3524 (2)	0.4554 (2)	0.0308 (9)	
H1A	0.2763	0.3082	0.4220	0.046*	
H1B	0.2502	0.3778	0.4827	0.046*	
H1C	0.3585	0.3308	0.4944	0.046*	
C2	0.4287 (3)	0.55396 (19)	0.4238 (2)	0.0271 (8)	
H2A	0.4204	0.5597	0.4803	0.041*	
H2B	0.3893	0.5965	0.3945	0.041*	
H2C	0.5022	0.5587	0.4149	0.041*	
C3	0.2257 (2)	0.50146 (18)	0.25852 (18)	0.0162 (7)	
C4	0.1430 (2)	0.55217 (18)	0.22049 (18)	0.0152 (7)	
C5	0.0645 (2)	0.58043 (18)	0.26549 (19)	0.0171 (7)	
H5	0.0652	0.5660	0.3196	0.021*	
C6	−0.0138 (2)	0.62907 (19)	0.23155 (18)	0.0181 (7)	
H6	−0.0666	0.6478	0.2626	0.022*	
C7	−0.0163 (2)	0.65090 (19)	0.15242 (19)	0.0181 (7)	
C8	0.0604 (2)	0.6219 (2)	0.10710 (19)	0.0215 (8)	
H8	0.0593	0.6360	0.0528	0.026*	
C9	0.1378 (2)	0.57307 (19)	0.14092 (19)	0.0202 (7)	
H9	0.1891	0.5530	0.1092	0.024*	
C10	0.4458 (2)	0.39988 (18)	0.21694 (18)	0.0160 (7)	
H10	0.4339	0.4055	0.1613	0.019*	
C11	0.5367 (2)	0.35692 (18)	0.24451 (19)	0.0169 (7)	
C12	0.5666 (3)	0.34850 (19)	0.32603 (19)	0.0190 (7)	
C13	0.6590 (3)	0.30561 (18)	0.3504 (2)	0.0224 (8)	
H13	0.6788	0.2995	0.4052	0.027*	
C14	0.7193 (3)	0.27336 (19)	0.2977 (2)	0.0226 (8)	
H14	0.7804	0.2450	0.3162	0.027*	
C15	0.6935 (3)	0.28078 (18)	0.2147 (2)	0.0193 (7)	
C16	0.7574 (3)	0.24726 (19)	0.1600 (2)	0.0259 (8)	
H16	0.8183	0.2187	0.1789	0.031*	
C17	0.7338 (3)	0.2550 (2)	0.0806 (2)	0.0334 (9)	
H17	0.7775	0.2318	0.0445	0.040*	
C18	0.6446 (3)	0.2973 (2)	0.0530 (2)	0.0365 (10)	
H18	0.6282	0.3035	−0.0022	0.044*	
C19	0.5802 (3)	0.3301 (2)	0.1050 (2)	0.0308 (9)	
H19	0.5194	0.3581	0.0849	0.037*	
C20	0.6022 (3)	0.32324 (18)	0.18701 (19)	0.0188 (7)	
Sn2	0.863063 (17)	0.698292 (13)	0.901481 (13)	0.01777 (7)	
O4	0.72509 (17)	0.75323 (13)	0.84463 (12)	0.0234 (5)	
O5	0.99479 (18)	0.62575 (14)	0.90442 (13)	0.0291 (6)	
O6	0.40545 (17)	0.94156 (14)	0.60703 (13)	0.0240 (5)	
H6A	0.3654	0.9612	0.6384	0.036*	
N3	0.79687 (19)	0.72937 (15)	0.72772 (15)	0.0150 (6)	
N4	0.8715 (2)	0.68727 (14)	0.77582 (15)	0.0150 (6)	
C21	0.7751 (3)	0.6187 (2)	0.96517 (19)	0.0261 (8)	
H21A	0.7460	0.5760	0.9299	0.039*	
H21B	0.7187	0.6486	0.9869	0.039*	
H21C	0.8196	0.5944	1.0084	0.039*	
C22	0.9403 (3)	0.80532 (19)	0.9414 (2)	0.0290 (9)	
H22A	0.9540	0.8034	0.9990	0.043*	
H22B	0.8966	0.8523	0.9266	0.043*	
H22C	1.0061	0.8099	0.9171	0.043*	
C23	0.7252 (2)	0.76115 (18)	0.76921 (19)	0.0167 (7)	
C24	0.6415 (2)	0.80870 (17)	0.72659 (18)	0.0151 (7)	
C25	0.5548 (2)	0.83069 (18)	0.76511 (19)	0.0166 (7)	
H25	0.5506	0.8156	0.8187	0.020*	
C26	0.4749 (2)	0.87418 (18)	0.72596 (19)	0.0189 (7)	
H26	0.4157	0.8880	0.7526	0.023*	
C27	0.4805 (2)	0.89776 (19)	0.64805 (19)	0.0175 (7)	
C28	0.5677 (2)	0.8776 (2)	0.60977 (19)	0.0210 (8)	
H28	0.5731	0.8950	0.5570	0.025*	
C29	0.6457 (2)	0.83292 (19)	0.64771 (19)	0.0196 (7)	
H29	0.7037	0.8180	0.6203	0.023*	
C30	0.9417 (2)	0.65032 (17)	0.73749 (19)	0.0166 (7)	
H30	0.9319	0.6528	0.6815	0.020*	
C31	1.0306 (2)	0.60716 (18)	0.76933 (19)	0.0165 (7)	
C32	1.0538 (3)	0.59785 (19)	0.8512 (2)	0.0226 (8)	
C33	1.1456 (3)	0.55688 (19)	0.8799 (2)	0.0274 (8)	
H33	1.1609	0.5505	0.9353	0.033*	
C34	1.2119 (3)	0.52668 (19)	0.8300 (2)	0.0278 (9)	
H34	1.2737	0.5008	0.8511	0.033*	
C35	1.1910 (3)	0.53289 (18)	0.7467 (2)	0.0230 (8)	
C36	1.2606 (3)	0.49950 (19)	0.6957 (2)	0.0288 (9)	
H36	1.3221	0.4735	0.7171	0.035*	
C37	1.2394 (3)	0.5046 (2)	0.6157 (2)	0.0359 (10)	
H37	1.2868	0.4824	0.5818	0.043*	
C38	1.1497 (3)	0.5418 (2)	0.5836 (2)	0.0361 (10)	
H38	1.1354	0.5443	0.5279	0.043*	
C39	1.0809 (3)	0.5753 (2)	0.6322 (2)	0.0263 (8)	
H39	1.0195	0.6005	0.6092	0.032*	
C40	1.0997 (2)	0.57292 (18)	0.7152 (2)	0.0186 (7)	

### Atomic displacement parameters (Å^2^)

**Table:**

	*U*^11^	*U*^22^	*U*^33^	*U*^12^	*U*^13^	*U*^23^
Sn1	0.02143 (14)	0.02241 (13)	0.01285 (12)	−0.00068 (10)	0.00070 (10)	−0.00022 (10)
O1	0.0201 (13)	0.0329 (13)	0.0160 (12)	0.0038 (11)	0.0031 (10)	0.0017 (11)
O2	0.0300 (14)	0.0385 (14)	0.0150 (12)	0.0083 (12)	−0.0013 (11)	0.0023 (11)
O3	0.0174 (13)	0.0313 (13)	0.0193 (12)	0.0065 (11)	0.0023 (10)	−0.0044 (11)
N1	0.0128 (14)	0.0185 (14)	0.0163 (14)	0.0014 (12)	0.0005 (12)	−0.0022 (12)
N2	0.0137 (14)	0.0180 (14)	0.0151 (14)	−0.0010 (12)	−0.0027 (12)	0.0009 (12)
C1	0.042 (2)	0.032 (2)	0.0191 (19)	−0.0061 (18)	0.0068 (18)	−0.0007 (16)
C2	0.034 (2)	0.0242 (19)	0.0220 (19)	0.0000 (16)	−0.0013 (17)	−0.0006 (16)
C3	0.0127 (17)	0.0159 (16)	0.0196 (18)	−0.0071 (14)	−0.0001 (14)	−0.0061 (14)
C4	0.0114 (16)	0.0177 (16)	0.0163 (17)	−0.0039 (14)	0.0009 (13)	−0.0032 (14)
C5	0.0163 (17)	0.0201 (17)	0.0151 (17)	−0.0038 (14)	0.0022 (14)	−0.0006 (14)
C6	0.0142 (17)	0.0221 (17)	0.0190 (18)	−0.0016 (14)	0.0069 (14)	−0.0031 (15)
C7	0.0105 (17)	0.0215 (18)	0.0218 (18)	0.0012 (14)	−0.0015 (14)	−0.0024 (15)
C8	0.0175 (18)	0.0322 (19)	0.0148 (17)	0.0054 (16)	0.0018 (15)	−0.0033 (15)
C9	0.0146 (17)	0.0258 (18)	0.0206 (18)	0.0014 (15)	0.0033 (15)	−0.0063 (15)
C10	0.0180 (17)	0.0166 (16)	0.0137 (16)	−0.0015 (14)	0.0026 (14)	−0.0018 (14)
C11	0.0172 (18)	0.0144 (16)	0.0186 (17)	−0.0019 (14)	−0.0012 (14)	0.0004 (14)
C12	0.0191 (18)	0.0180 (17)	0.0197 (18)	−0.0006 (14)	0.0008 (15)	0.0003 (14)
C13	0.0223 (19)	0.0219 (18)	0.0219 (18)	−0.0035 (15)	−0.0053 (16)	0.0062 (15)
C14	0.0185 (19)	0.0170 (17)	0.031 (2)	0.0036 (15)	−0.0045 (16)	0.0066 (16)
C15	0.0173 (18)	0.0120 (16)	0.0276 (19)	−0.0004 (14)	−0.0037 (15)	0.0018 (15)
C16	0.0186 (19)	0.0184 (18)	0.040 (2)	0.0060 (15)	0.0003 (17)	−0.0025 (17)
C17	0.032 (2)	0.035 (2)	0.034 (2)	0.0126 (18)	0.0064 (19)	−0.0082 (18)
C18	0.041 (2)	0.050 (2)	0.0190 (19)	0.019 (2)	0.0012 (18)	−0.0046 (18)
C19	0.026 (2)	0.042 (2)	0.024 (2)	0.0161 (18)	−0.0010 (17)	−0.0065 (18)
C20	0.0185 (18)	0.0159 (17)	0.0213 (18)	−0.0012 (14)	−0.0020 (15)	−0.0028 (14)
Sn2	0.01788 (13)	0.02101 (13)	0.01475 (12)	0.00164 (10)	0.00322 (10)	0.00117 (10)
O4	0.0200 (13)	0.0354 (14)	0.0157 (12)	0.0102 (11)	0.0067 (10)	0.0035 (11)
O5	0.0279 (14)	0.0394 (14)	0.0200 (13)	0.0163 (12)	0.0025 (11)	0.0036 (11)
O6	0.0190 (13)	0.0342 (14)	0.0188 (12)	0.0109 (11)	0.0010 (10)	−0.0031 (11)
N3	0.0095 (14)	0.0184 (14)	0.0172 (14)	0.0025 (11)	0.0012 (11)	0.0008 (12)
N4	0.0137 (14)	0.0156 (13)	0.0157 (14)	0.0007 (11)	0.0016 (12)	0.0026 (11)
C21	0.036 (2)	0.0276 (19)	0.0147 (18)	−0.0106 (17)	0.0039 (16)	−0.0008 (15)
C22	0.028 (2)	0.028 (2)	0.031 (2)	−0.0085 (17)	0.0023 (18)	−0.0017 (17)
C23	0.0131 (17)	0.0148 (16)	0.0232 (18)	−0.0054 (14)	0.0072 (15)	−0.0013 (14)
C24	0.0144 (17)	0.0118 (15)	0.0195 (17)	−0.0017 (13)	0.0037 (14)	−0.0022 (14)
C25	0.0155 (17)	0.0162 (16)	0.0186 (17)	−0.0018 (14)	0.0035 (14)	−0.0024 (14)
C26	0.0133 (17)	0.0179 (17)	0.0263 (19)	−0.0028 (14)	0.0071 (15)	−0.0053 (15)
C27	0.0112 (17)	0.0197 (17)	0.0211 (18)	−0.0016 (14)	−0.0022 (14)	−0.0044 (15)
C28	0.0192 (18)	0.0302 (19)	0.0139 (17)	0.0025 (16)	0.0024 (14)	0.0002 (15)
C29	0.0140 (17)	0.0231 (17)	0.0225 (18)	0.0042 (15)	0.0067 (15)	−0.0061 (15)
C30	0.0188 (18)	0.0137 (16)	0.0180 (17)	−0.0041 (14)	0.0048 (15)	−0.0020 (14)
C31	0.0101 (16)	0.0165 (17)	0.0233 (18)	−0.0013 (13)	0.0038 (14)	−0.0008 (14)
C32	0.0220 (19)	0.0182 (17)	0.028 (2)	0.0012 (15)	0.0057 (16)	0.0027 (16)
C33	0.026 (2)	0.027 (2)	0.029 (2)	0.0046 (16)	−0.0004 (17)	0.0086 (17)
C34	0.021 (2)	0.0175 (17)	0.045 (2)	0.0058 (15)	0.0026 (18)	0.0093 (17)
C35	0.0171 (18)	0.0103 (16)	0.042 (2)	−0.0036 (14)	0.0062 (17)	0.0002 (16)
C36	0.019 (2)	0.0135 (17)	0.055 (3)	0.0025 (15)	0.0099 (19)	−0.0043 (18)
C37	0.030 (2)	0.028 (2)	0.053 (3)	0.0003 (18)	0.021 (2)	−0.018 (2)
C38	0.034 (2)	0.040 (2)	0.036 (2)	0.0026 (19)	0.012 (2)	−0.0114 (19)
C39	0.0194 (19)	0.0294 (19)	0.031 (2)	0.0020 (16)	0.0087 (17)	−0.0072 (17)
C40	0.0141 (17)	0.0136 (16)	0.0286 (19)	−0.0001 (14)	0.0051 (15)	−0.0024 (15)

### Geometric parameters (Å, °)

**Table:**

Sn1—O2	2.083 (2)	Sn2—O5	2.081 (2)
Sn1—C1	2.112 (3)	Sn2—C21	2.099 (3)
Sn1—C2	2.116 (3)	Sn2—C22	2.115 (3)
Sn1—N2	2.152 (2)	Sn2—N4	2.155 (2)
Sn1—O1	2.159 (2)	Sn2—O4	2.157 (2)
O1—C3	1.297 (4)	O4—C23	1.288 (4)
O2—C12	1.316 (4)	O5—C32	1.316 (4)
O3—C7	1.355 (4)	O6—C27	1.355 (4)
O3—H3	0.8400	O6—H6A	0.8400
N1—C3	1.326 (4)	N3—C23	1.321 (4)
N1—N2	1.397 (3)	N3—N4	1.396 (3)
N2—C10	1.303 (4)	N4—C30	1.314 (4)
C1—H1A	0.9800	C21—H21A	0.9800
C1—H1B	0.9800	C21—H21B	0.9800
C1—H1C	0.9800	C21—H21C	0.9800
C2—H2A	0.9800	C22—H22A	0.9800
C2—H2B	0.9800	C22—H22B	0.9800
C2—H2C	0.9800	C22—H22C	0.9800
C3—C4	1.466 (4)	C23—C24	1.477 (4)
C4—C9	1.392 (4)	C24—C25	1.395 (4)
C4—C5	1.403 (4)	C24—C29	1.405 (4)
C5—C6	1.380 (4)	C25—C26	1.382 (4)
C5—H5	0.9500	C25—H25	0.9500
C6—C7	1.390 (4)	C26—C27	1.388 (4)
C6—H6	0.9500	C26—H26	0.9500
C7—C8	1.393 (4)	C27—C28	1.390 (4)
C8—C9	1.373 (4)	C28—C29	1.367 (4)
C8—H8	0.9500	C28—H28	0.9500
C9—H9	0.9500	C29—H29	0.9500
C10—C11	1.418 (4)	C30—C31	1.421 (4)
C10—H10	0.9500	C30—H30	0.9500
C11—C12	1.413 (4)	C31—C32	1.407 (5)
C11—C20	1.458 (4)	C31—C40	1.452 (4)
C12—C13	1.421 (4)	C32—C33	1.417 (5)
C13—C14	1.347 (4)	C33—C34	1.352 (4)
C13—H13	0.9500	C33—H33	0.9500
C14—C15	1.427 (4)	C34—C35	1.423 (5)
C14—H14	0.9500	C34—H34	0.9500
C15—C16	1.409 (4)	C35—C36	1.415 (4)
C15—C20	1.419 (4)	C35—C40	1.418 (5)
C16—C17	1.364 (5)	C36—C37	1.365 (5)
C16—H16	0.9500	C36—H36	0.9500
C17—C18	1.395 (5)	C37—C38	1.384 (5)
C17—H17	0.9500	C37—H37	0.9500
C18—C19	1.377 (5)	C38—C39	1.381 (5)
C18—H18	0.9500	C38—H38	0.9500
C19—C20	1.402 (5)	C39—C40	1.410 (5)
C19—H19	0.9500	C39—H39	0.9500
			
O2—Sn1—C1	95.19 (12)	O5—Sn2—C21	96.08 (12)
O2—Sn1—C2	99.24 (12)	O5—Sn2—C22	96.34 (12)
C1—Sn1—C2	125.14 (13)	C21—Sn2—C22	128.37 (13)
O2—Sn1—N2	81.92 (9)	O5—Sn2—N4	82.19 (9)
C1—Sn1—N2	125.45 (11)	C21—Sn2—N4	122.25 (11)
C2—Sn1—N2	108.92 (11)	C22—Sn2—N4	109.01 (12)
O2—Sn1—O1	154.21 (8)	O5—Sn2—O4	153.95 (9)
C1—Sn1—O1	94.20 (12)	C21—Sn2—O4	91.72 (12)
C2—Sn1—O1	94.83 (11)	C22—Sn2—O4	98.16 (12)
N2—Sn1—O1	73.08 (9)	N4—Sn2—O4	72.67 (9)
C3—O1—Sn1	113.32 (19)	C23—O4—Sn2	114.62 (19)
C12—O2—Sn1	135.1 (2)	C32—O5—Sn2	135.1 (2)
C7—O3—H3	109.5	C27—O6—H6A	109.5
C3—N1—N2	112.2 (3)	C23—N3—N4	111.6 (2)
C10—N2—N1	115.0 (3)	C30—N4—N3	114.5 (3)
C10—N2—Sn1	129.4 (2)	C30—N4—Sn2	128.8 (2)
N1—N2—Sn1	115.33 (17)	N3—N4—Sn2	116.53 (17)
Sn1—C1—H1A	109.5	Sn2—C21—H21A	109.5
Sn1—C1—H1B	109.5	Sn2—C21—H21B	109.5
H1A—C1—H1B	109.5	H21A—C21—H21B	109.5
Sn1—C1—H1C	109.5	Sn2—C21—H21C	109.5
H1A—C1—H1C	109.5	H21A—C21—H21C	109.5
H1B—C1—H1C	109.5	H21B—C21—H21C	109.5
Sn1—C2—H2A	109.5	Sn2—C22—H22A	109.5
Sn1—C2—H2B	109.5	Sn2—C22—H22B	109.5
H2A—C2—H2B	109.5	H22A—C22—H22B	109.5
Sn1—C2—H2C	109.5	Sn2—C22—H22C	109.5
H2A—C2—H2C	109.5	H22A—C22—H22C	109.5
H2B—C2—H2C	109.5	H22B—C22—H22C	109.5
O1—C3—N1	122.9 (3)	O4—C23—N3	123.5 (3)
O1—C3—C4	119.5 (3)	O4—C23—C24	118.6 (3)
N1—C3—C4	117.5 (3)	N3—C23—C24	118.0 (3)
C9—C4—C5	118.1 (3)	C25—C24—C29	118.2 (3)
C9—C4—C3	122.9 (3)	C25—C24—C23	119.6 (3)
C5—C4—C3	119.1 (3)	C29—C24—C23	122.2 (3)
C6—C5—C4	120.4 (3)	C26—C25—C24	120.6 (3)
C6—C5—H5	119.8	C26—C25—H25	119.7
C4—C5—H5	119.8	C24—C25—H25	119.7
C5—C6—C7	120.7 (3)	C25—C26—C27	120.5 (3)
C5—C6—H6	119.7	C25—C26—H26	119.8
C7—C6—H6	119.7	C27—C26—H26	119.8
O3—C7—C6	123.0 (3)	O6—C27—C26	123.1 (3)
O3—C7—C8	117.8 (3)	O6—C27—C28	117.5 (3)
C6—C7—C8	119.2 (3)	C26—C27—C28	119.3 (3)
C9—C8—C7	120.0 (3)	C29—C28—C27	120.3 (3)
C9—C8—H8	120.0	C29—C28—H28	119.8
C7—C8—H8	120.0	C27—C28—H28	119.8
C8—C9—C4	121.7 (3)	C28—C29—C24	121.1 (3)
C8—C9—H9	119.2	C28—C29—H29	119.4
C4—C9—H9	119.2	C24—C29—H29	119.4
N2—C10—C11	128.2 (3)	N4—C30—C31	128.1 (3)
N2—C10—H10	115.9	N4—C30—H30	116.0
C11—C10—H10	115.9	C31—C30—H30	116.0
C12—C11—C10	121.5 (3)	C32—C31—C30	121.9 (3)
C12—C11—C20	119.5 (3)	C32—C31—C40	119.6 (3)
C10—C11—C20	118.9 (3)	C30—C31—C40	118.5 (3)
O2—C12—C11	123.6 (3)	O5—C32—C31	123.6 (3)
O2—C12—C13	117.2 (3)	O5—C32—C33	116.7 (3)
C11—C12—C13	119.2 (3)	C31—C32—C33	119.6 (3)
C14—C13—C12	121.7 (3)	C34—C33—C32	121.2 (3)
C14—C13—H13	119.2	C34—C33—H33	119.4
C12—C13—H13	119.2	C32—C33—H33	119.4
C13—C14—C15	121.6 (3)	C33—C34—C35	121.4 (3)
C13—C14—H14	119.2	C33—C34—H34	119.3
C15—C14—H14	119.2	C35—C34—H34	119.3
C16—C15—C20	119.7 (3)	C36—C35—C40	120.3 (3)
C16—C15—C14	121.1 (3)	C36—C35—C34	120.3 (3)
C20—C15—C14	119.1 (3)	C40—C35—C34	119.4 (3)
C17—C16—C15	121.4 (3)	C37—C36—C35	120.1 (4)
C17—C16—H16	119.3	C37—C36—H36	119.9
C15—C16—H16	119.3	C35—C36—H36	119.9
C16—C17—C18	119.2 (3)	C36—C37—C38	120.6 (3)
C16—C17—H17	120.4	C36—C37—H37	119.7
C18—C17—H17	120.4	C38—C37—H37	119.7
C19—C18—C17	120.7 (3)	C39—C38—C37	120.3 (4)
C19—C18—H18	119.7	C39—C38—H38	119.8
C17—C18—H18	119.7	C37—C38—H38	119.8
C18—C19—C20	121.5 (3)	C38—C39—C40	121.4 (4)
C18—C19—H19	119.2	C38—C39—H39	119.3
C20—C19—H19	119.2	C40—C39—H39	119.3
C19—C20—C15	117.5 (3)	C39—C40—C35	117.2 (3)
C19—C20—C11	123.7 (3)	C39—C40—C31	124.1 (3)
C15—C20—C11	118.8 (3)	C35—C40—C31	118.7 (3)
			
O2—Sn1—O1—C3	−29.7 (3)	O5—Sn2—O4—C23	−24.6 (3)
C1—Sn1—O1—C3	−140.9 (2)	C21—Sn2—O4—C23	−132.3 (2)
C2—Sn1—O1—C3	93.3 (2)	C22—Sn2—O4—C23	98.6 (2)
N2—Sn1—O1—C3	−15.0 (2)	N4—Sn2—O4—C23	−8.9 (2)
C1—Sn1—O2—C12	120.3 (3)	C21—Sn2—O5—C32	128.0 (3)
C2—Sn1—O2—C12	−112.8 (3)	C22—Sn2—O5—C32	−102.2 (3)
N2—Sn1—O2—C12	−4.8 (3)	N4—Sn2—O5—C32	6.2 (3)
O1—Sn1—O2—C12	9.4 (4)	O4—Sn2—O5—C32	21.3 (4)
C3—N1—N2—C10	173.8 (3)	C23—N3—N4—C30	176.3 (2)
C3—N1—N2—Sn1	−12.0 (3)	C23—N3—N4—Sn2	−7.9 (3)
O2—Sn1—N2—C10	1.2 (3)	O5—Sn2—N4—C30	−2.8 (2)
C1—Sn1—N2—C10	−89.4 (3)	C21—Sn2—N4—C30	−95.1 (3)
C2—Sn1—N2—C10	98.3 (3)	C22—Sn2—N4—C30	91.2 (3)
O1—Sn1—N2—C10	−172.4 (3)	O4—Sn2—N4—C30	−175.9 (3)
O2—Sn1—N2—N1	−172.0 (2)	O5—Sn2—N4—N3	−177.9 (2)
C1—Sn1—N2—N1	97.4 (2)	C21—Sn2—N4—N3	89.8 (2)
C2—Sn1—N2—N1	−74.9 (2)	C22—Sn2—N4—N3	−83.9 (2)
O1—Sn1—N2—N1	14.45 (18)	O4—Sn2—N4—N3	8.99 (19)
Sn1—O1—C3—N1	14.9 (4)	Sn2—O4—C23—N3	8.4 (4)
Sn1—O1—C3—C4	−163.7 (2)	Sn2—O4—C23—C24	−171.2 (2)
N2—N1—C3—O1	−2.1 (4)	N4—N3—C23—O4	−0.4 (4)
N2—N1—C3—C4	176.5 (2)	N4—N3—C23—C24	179.1 (2)
O1—C3—C4—C9	179.9 (3)	O4—C23—C24—C25	−12.1 (4)
N1—C3—C4—C9	1.3 (5)	N3—C23—C24—C25	168.4 (3)
O1—C3—C4—C5	−0.8 (4)	O4—C23—C24—C29	168.0 (3)
N1—C3—C4—C5	−179.4 (3)	N3—C23—C24—C29	−11.5 (4)
C9—C4—C5—C6	−1.5 (5)	C29—C24—C25—C26	0.8 (5)
C3—C4—C5—C6	179.3 (3)	C23—C24—C25—C26	−179.1 (3)
C4—C5—C6—C7	0.0 (5)	C24—C25—C26—C27	−1.0 (5)
C5—C6—C7—O3	−179.4 (3)	C25—C26—C27—O6	−178.9 (3)
C5—C6—C7—C8	1.1 (5)	C25—C26—C27—C28	−0.4 (5)
O3—C7—C8—C9	179.9 (3)	O6—C27—C28—C29	−179.4 (3)
C6—C7—C8—C9	−0.6 (5)	C26—C27—C28—C29	2.0 (5)
C7—C8—C9—C4	−0.9 (5)	C27—C28—C29—C24	−2.2 (5)
C5—C4—C9—C8	2.0 (5)	C25—C24—C29—C28	0.8 (5)
C3—C4—C9—C8	−178.8 (3)	C23—C24—C29—C28	−179.3 (3)
N1—N2—C10—C11	175.5 (3)	N3—N4—C30—C31	175.7 (3)
Sn1—N2—C10—C11	2.3 (5)	Sn2—N4—C30—C31	0.6 (5)
N2—C10—C11—C12	−3.8 (5)	N4—C30—C31—C32	0.9 (5)
N2—C10—C11—C20	178.3 (3)	N4—C30—C31—C40	−178.2 (3)
Sn1—O2—C12—C11	4.8 (5)	Sn2—O5—C32—C31	−6.8 (5)
Sn1—O2—C12—C13	−175.5 (2)	Sn2—O5—C32—C33	172.8 (2)
C10—C11—C12—O2	0.3 (5)	C30—C31—C32—O5	2.0 (5)
C20—C11—C12—O2	178.2 (3)	C40—C31—C32—O5	−178.9 (3)
C10—C11—C12—C13	−179.3 (3)	C30—C31—C32—C33	−177.6 (3)
C20—C11—C12—C13	−1.5 (4)	C40—C31—C32—C33	1.5 (5)
O2—C12—C13—C14	−179.1 (3)	O5—C32—C33—C34	−179.4 (3)
C11—C12—C13—C14	0.6 (5)	C31—C32—C33—C34	0.3 (5)
C12—C13—C14—C15	0.1 (5)	C32—C33—C34—C35	−1.6 (5)
C13—C14—C15—C16	179.6 (3)	C33—C34—C35—C36	−178.8 (3)
C13—C14—C15—C20	0.1 (5)	C33—C34—C35—C40	1.2 (5)
C20—C15—C16—C17	0.3 (5)	C40—C35—C36—C37	−1.0 (5)
C14—C15—C16—C17	−179.2 (3)	C34—C35—C36—C37	179.1 (3)
C15—C16—C17—C18	0.3 (6)	C35—C36—C37—C38	−0.4 (5)
C16—C17—C18—C19	−0.8 (6)	C36—C37—C38—C39	0.8 (6)
C17—C18—C19—C20	0.8 (6)	C37—C38—C39—C40	0.2 (6)
C18—C19—C20—C15	−0.2 (5)	C38—C39—C40—C35	−1.6 (5)
C18—C19—C20—C11	179.9 (3)	C38—C39—C40—C31	179.8 (3)
C16—C15—C20—C19	−0.3 (5)	C36—C35—C40—C39	1.9 (5)
C14—C15—C20—C19	179.2 (3)	C34—C35—C40—C39	−178.1 (3)
C16—C15—C20—C11	179.5 (3)	C36—C35—C40—C31	−179.4 (3)
C14—C15—C20—C11	−0.9 (4)	C34—C35—C40—C31	0.6 (4)
C12—C11—C20—C19	−178.5 (3)	C32—C31—C40—C39	176.7 (3)
C10—C11—C20—C19	−0.6 (5)	C30—C31—C40—C39	−4.2 (5)
C12—C11—C20—C15	1.6 (4)	C32—C31—C40—C35	−1.9 (4)
C10—C11—C20—C15	179.5 (3)	C30—C31—C40—C35	177.2 (3)

### Hydrogen-bond geometry (Å, °)

**Table:**

*D*—H···*A*	*D*—H	H···*A*	*D*···*A*	*D*—H···*A*
O3—H3···N3^i^	0.84	1.91	2.749 (3)	178
O6—H6a···N1^ii^	0.84	1.91	2.738 (3)	167
C8—H8···O5^iii^	0.95	2.59	3.477 (4)	155
C21—H21a···N1^iv^	0.98	2.62	3.494 (4)	149

Symmetry codes: (i) *x*−1, −*y*+3/2, *z*−1/2; (ii) *x*, −*y*+3/2, *z*+1/2; (iii) *x*−1, *y*, *z*−1; (iv) −*x*+1, −*y*+1, −*z*+1.
